# COVID-19 and food insecurity in a vulnerable rural state

**DOI:** 10.1016/j.dialog.2022.100013

**Published:** 2022-05-13

**Authors:** Don E. Willis, Christopher R. Long, Brett Rowland, Caitlin Tidwell, Jennifer A. Andersen, Pearl A. McElfish

**Affiliations:** a University of Arkansas for Medical Sciences Northwest, College of Medicine, 1125 N. College Avenue, Fayetteville, AR 72703, USA; b University of Arkansas for Medical Sciences Northwest, Office of Community Health and Research, 1125 N. College Avenue, Fayetteville, AR 72703, USA

**Keywords:** Food insecurity, COVID-19, Income, Housing, Public health nutrition, Pandemic

## Abstract

**Objective:**

This study explored variations in food insecurity across sociodemographic groups and changes specific to the COVID-19 pandemic, including income loss, stimulus check receipt, and changes in household size.

**Design:**

A cross-sectional online survey was conducted using a 2-item food insecurity screener. COVID-19 related factors and sociodemographic data were collected.

**Setting:**

Data were collected in Arkansas, United States, during July and August 2020.

**Participants:**

A sample of 1205 adults was recruited using ARresearch, a volunteer research registry. Participants were over the age of 18 and living, working, or receiving health care in Arkansas.

**Results:**

The prevalence of food insecurity was 24.9% during the COVID-19 pandemic. Food insecurity was elevated even after the majority of respondents received a stimulus check. Chi-square and *t*-tests revealed that food insecurity was more prevalent among those who are younger, Black, Hispanic/Latinx, lower-income, less educated, and living in households with children. Multivariate logistic regression revealed that odds of food insecurity were greater for individuals who reported income loss due to the pandemic (OR = 3.29; *p* < .001), Black respondents (OR = 2.06, *p* = .014), Hispanic respondents (OR = 3.34, *p* = .001), those earning less than $25,000 annually (OR = 4.92; *p* < .001) or between $25,000 to $49,999 (OR = 2.04; *p* = .023), respondents with a high school degree or less (OR = 4.21; *p* < .001) or some college (OR = 2.55; *p* < .001), and those living in households with children (OR = 1.62; *p* = .021). Odds of food insecurity were lower for those who had received a stimulus check (OR = 0.60; *p* = .026).

**Conclusion:**

Food insecurity prevalence was high in Arkansas in July and August 2020. The risk of food insecurity was uneven across sociodemographic groups. Several factors related to the COVID-19 pandemic were indicators for increased risk of food insecurity. Interventions to address food insecurity that recognize social factors unique to the pandemic are needed to reduce levels of food insecurity.

## 1 Introduction

The first cases of COVID-19 were identified in the United States (US) in early 2020. By the end of 2020, COVID-19 was the leading cause of death in the US [1], taking more than 400,000 lives [2] and disproportionately killing people of color [[3], [4], [5], [6], [7]]. The effects of the pandemic have not been limited to those infected by the COVID-19 virus; it has also had a negative economic impact. By the end of March 2020, more than 700,000 jobs had been lost, and unemployment rose to 7.1 million [8]. The COVID-19 pandemic prompted widespread social and policy changes that led to high rates of unemployment and income loss [9]. By April of 2020, 43% of US adults reported they or someone in their household had lost a job or taken a pay cut because of COVID-19 [10]. Households with children (55.0%) and lower-income adults (52.0%) reported the highest rates of income loss [9,11]. Food insecurity within the general population rose to 38.3% [12], with more than a third (35.0%) of the food insecure reporting they were “newly food insecure” [13].

While research has documented higher food insecurity during COVID-19, studies have not identified the sociodemographic factors associated with food insecurity during the pandemic, and the literature has not examined COVID-19-related experiences and their influence on food insecurity. The objective of this study was to explore variations in food insecurity by examining sociodemographic factors and changes in income loss, stimulus check receipt, and changes in household size during the COVID-19 pandemic. We focus analysis on Arkansas because Arkansas has consistently been one of the most food insecure states in the US [14], and Arkansas' already-high food insecurity rate was estimated to increase during the COVID-19 pandemic [15].

The present study asked the following research questions: 1) How prevalent was food insecurity in Arkansas during the pandemic (July/August 2020) (after stimulus checks had been received by a large portion of the US population)? 2) Which groups of adult Arkansans had the highest prevalence of food insecurity during this period of the pandemic? 3) What COVID-19-related factors (e.g., income loss, stimulus receipt, changes in household size) are associated with food insecurity? Answering these questions will be critical to understanding which populations were most likely to experience food insecurity at this time during the pandemic and what pandemic-related factors may have increased or reduced odds of experiencing food insecurity.

## 2 Methods

Respondents were recruited in July/August of 2020 through ARresearch [16], a volunteer research registry operated by the University of Arkansas for Medical Sciences. The ARresearch registry closely mirrors the racial and ethnic composition of Arkansas. Potential participants were sent an email describing the study and were provided the opportunity to document their consent. Respondents had to be over the age of 18 and living, working, or receiving health care in the state of Arkansas to be eligible for inclusion in the study. Data was collected using REDCap, a web-based software for data capture and management [17]. Respondents were provided a $20 gift card for completion of the survey. The study was reviewed and approved by the Institutional Review Board at the University of Arkansas for Medical Sciences (IRB#261226).

### 2.1 Measurement

The survey captured sociodemographic characteristics, including age, sex, race, income, education, and whether or not children lived in the household. Food insecurity was measured using a validated 2-item screener, modified to focus on the past 30 days rather than the standard 12 months [18]. Respondents were considered food insecure if they responded affirmatively to either of the following two items: “Within the past 30 days, we worried whether our food would run out before we got money to buy more.” and “Within the past 30 days, the food we bought just didn't last and we didn't have money to get more.” Participants were asked if they had lost income due to the pandemic, if they had received a stimulus check, and whether the size of their household changed (i.e., increases or decreases in the number of people living in the household) during the pandemic.

### 2.2 Analysis

We present basic descriptive statistics for the sample. We present chi-square and *t*-tests to analyze bivariate associations between food insecurity and the following independent variables: age, sex, race, income, education, children in the household, income loss, receipt of a stimulus payment, and changes in household size since the pandemic began. We present results from a logistic regression to analyze associations with odds of food insecurity while adjusting for all independent variables (age, sex, race, income, education, children in the household, income loss, receipt of a stimulus payment, and changes in household size) in the model. Analyses were conducted with Stata 15 [19].

## 3 Results

A total of 1288 responses were collected during the survey period. Of those, 1221 met the age and location eligibility requirements; 16 eligible participants did not answer questions beyond the eligibility screener. An analytical sample of 1205 included all eligible participants who answered questions beyond the eligibility section of the survey.

Table 1 presents descriptive statistics for the sample as well as bivariate analyses for food insecurity and sociodemographic factors. Respondents' mean age was 48 years old (SD = 15.6), and three of four respondents were women (75.2%). One in four (24.9%) respondents were food insecure. Food insecure respondents were a mean of eleven years younger than their food secure counterparts (40.6 vs. 51.6; t(986) = 9.9; *p* < .001). We found no differences in food insecurity across sex (χ^2^(1) = 0.2; *p* = .688).

**Table 1:** Food insecurity among Arkansas adults during July/August 2020, *n* = 1205.

	Total n (%)	Food Secure n (%)	Food Insecure n (%)	*p*
Age (years, Mean ± SD)	48.1 ± 15.6	51.6 ± 15.8	40.6 ± 12.8	**<0.001**
Sex				0.688
Women	905(75.2)	550(74.7)	186(25.3)	
Men	298(24.8)	190(76.0)	60(24.0)	
Race/Ethnicity				**<0.001**
Black	161(13.4)	65(59.6)	44(40.4)	
White	918(76.5)	633(80.7)	151(19.3)	
Other	41(3.4)	22(62.9)	13(37.1)	
Hispanic/Latinx	80(6.7)	18(32.1)	38(67.9)	
Income				**<0.001**
< $25,000	205(21.1)	96(50.5)	94(49.5)	
$25,000 to < $50,000	284(29.2)	186(69.7)	81(30.3)	
$50,000 to < $75,000	207(22.7)	175(84.5)	32(15.5)	
$75,000+	264(27.1)	230(90.2)	25(9.8)	
Education				**<0.001**
HS degree or less	145(12.1)	51(46.0)	60(54.1)	
Some college	331(27.5)	165(62.0)	101(38.0)	
College degree or more	726(60.4)	526(86.2)	84(13.8)	
Household with Children				**<0.001**
Yes	371(30.8)	216(63.0)	127(37.0)	
No	834(69.2)	526(81.6)	119(18.5)	

Notes: SD = standard deviation; HS = high school.

Percentages may not total 100 due to rounding.

Comparisons between food secure and food insecure groups were made using t-test for age; all other comparisons were made using chi-square tests.

The sample was 13.4% non-Hispanic Black, 76.5% non-Hispanic White, 3.4% non-Hispanic people of other racial groups (e.g., Asian, American Indian or Alaska Native, Native Hawaiian or Pacific Islander), and 6.7% Hispanic or Latinx residents of any race. The sample closely mirrored 2019 American Community Survey estimates of racial categories for Arkansas: 15.4% non-Hispanic Black, 72.0% non-Hispanic White, 4.8% non-Hispanic people of other racial or ethnic groups, and 7.7% Hispanic or Latinx residents of any race. Food insecurity was higher among Black (40.4), Hispanic/Latinx (67.9), and other racial groups (37.1) compared to White respondents (19.3). These differences were statistically significant (χ^2^(3) = 85.1; *p* < .001).

Approximately one third of respondents earned between $25,000 to $49,999 annually (29.2%), followed by those earning $75,000 or more (27.1%), those making $50,000 to $74,999 (22.7%), and those making less than $25,000 (21.1%). Food insecurity was significantly associated with income (χ^2^(3) = 105.5; *p* < .001), with 9.8% of those in the highest income category and 49.5% in the lowest income category experiencing food insecurity.

The majority of the sample held a four-year college degree (60.4%), and 13.8% of college degree holders reported food insecurity. Food insecurity was significantly associated with educational attainment (χ^2^(2) = 115.4; *p* < .001) and was most prevalent among those with some college (38.0%) or a high school degree or less (54.1%), compared to much lower prevalence among those with a college degree (13.8%).

Households with children comprised 30.8% of the sample and had a higher prevalence of food insecurity (37.0%) compared to those who did not have children (18.5%) (χ^2^(1) = 41.3; *p* < .001).

Table 2 presents bivariate analyses of food insecurity and changes specific to COVID-19. More than a third (36.2%) of the sample reported they had lost income since the pandemic started. Of those who lost income, 43.9% were food insecure compared to 13.8% who reported no income loss. This difference was statistically significant (χ^2^(1) = 106.5; *p* < .001).

**Table 2:** COVID-19 changes and food insecurity for Arkansas adults, *n* = 1205.

	Total n (%)	Food Secure n (%)	Food Insecure n (%)	*p*
COVID-19 Income Loss				**<0.001**
Yes	361 (36.2)	188 (56.1)	147 (43.9)	
No	637 (63.8)	527 (86.3)	84 (13.8)	
Stimulus Payment				**<0.001**
Received	833 (80.1)	624 (79.4)	162 (20.6)	
Not received/eligible	207 (19.9)	114 (59.4)	78 (40.6)	
Change in Household Size				**<0.05**
Increased/Decreased	111 (10.5)	68 (66.7)	34 (33.3)	
Stable	946 (89.5)	670 (76.1)	210 (23.9)	

Notes: Percentages may not total 100 due to rounding.

All comparisons between food secure and food insecure groups were made using chi-square tests.

Four in five (80.1%) respondents had received a stimulus check at the time of the survey. One in five (20.6%) respondents who had received a stimulus check reported food insecurity, while two in five (40.6%) who had not received a stimulus check were food insecure. This difference was statistically significant (χ^2^(1) = 33.4; *p* < .001).

One in ten (10.5%) respondents experienced changes in their household size since the pandemic began. A third (33.3%) of respondents who experienced changes in their household size were food insecure, while 23.9% of those whose household composition was stable were food insecure. This difference was statistically significant (χ^2^(1) = 4.4; *p* < .05).

### 3.1 Multivariate model

Table 3 presents results of a multivariate logistic regression analysis examining odds ratios for sociodemographic characteristics as well as COVID-19-related changes experienced by respondents. We find significant associations between odds of food insecurity and age, race/ethnicity, income, education, households with children, income loss due to COVID-19, and receiving a stimulus check. Odds of food insecurity decreased as age increased (OR = 0.98; *p* = .001). Black respondents had 2.06 greater odds of food insecurity compared to White respondents (*p* = .014). Hispanic/Latinx respondents had 3.34 greater odds of food insecurity compared to White respondents (*p* = .001). Respondents earning less than $25,000 annually had 4.92 greater odds of food insecurity compared to respondents earning over $75,000 (*p* < .001). Respondents earning between $25,000 to $49,999 annually had 2.04 greater odds of food insecurity compared to respondents earning over $75,000 (*p* = .023). No statistically significant differences were observed between the top two income categories (*p* = .239). Respondents with a high school degree or less had 4.21 greater odds of food insecurity than those with a four-year college degree (*p* < .001). Respondents with some college had 2.55 greater odds of food insecurity than those with a four-year college degree (*p* < .001). Respondents living in households with children had 1.62 greater odds of food insecurity compared to those with no children (*p* = .021). Respondents who reported a loss of income due to COVID-19 had 3.29 greater odds of food insecurity compared to those who had not lost income (*p* < .001). Those who had received a stimulus check had reduced odds of food insecurity compared to those who had not (OR = 0.60; *p* = .026). Change in household size, while significant in bivariate analysis, was not significantly associated with food insecurity after adjusting for sociodemographic characteristics and the COVID-19-related changes (*p* = .335).

**Table 3:** Associations between food insecurity and select characteristics of Arkansas adults: results of multivariate logistic regression, *n* = 867.

	*OR*	*SE*	95% CI	*p*
Age	0.98	0.01	(0.96, 0.99)	**0.001**
Sex				
Women	0.91	0.23	(0.58, 1.42)	0.675
Men	–	–	–	–
Race/Ethnicity				
Black	2.06	0.29	(1.16, 3.68)	**0.014**
Hispanic/Latinx	3.34	0.37	(1.62, 6.92)	**0.001**
Other	2.01	0.47	(0.81, 5.04)	0.135
White	–	–	–	**–**
Income				
< $25,000	4.92	0.33	(2.56, 9.46)	**<0.001**
$25,000 to < $50,000	2.04	0.31	(1.10, 3.78)	**0.023**
$50,000 to < $75,000	1.47	0.33	(0.78, 2.78)	0.239
$75,000+	–	–	–	–
Education				
HS degree or less	4.21	0.30	(2.34, 7.58)	**<0.001**
Some college	2.55	0.24	(1.60, 4.06)	**<0.001**
College degree or more	–	–	–	–
Household with Children				
Yes	1.62	0.21	(1.08, 2.44)	**0.021**
No	–	–	–	**–**
COVID-19 Income Loss				
Yes	3.29	0.20	(2.21, 4.88)	**<0.001**
No	–	–	–	**–**
Received Stimulus Payment				
Yes	0.60	0.23	(0.38, 0.94)	**0.026**
No	–	–	–	**–**
Change in Household Size				
Yes	1.33	0.29	(0.75, 2.36)	0.335
No	–	–	–	**–**
Constant	0.18	0.10	(0.06, 0.51)	0.**001**
Pseudo R^2^	0.293			

Notes: OR = odds ratio; SE = standard error; CI = confidence intervals; HS = high school.

## 4 Discussion

The present study found elevated food insecurity (24.9%) among a racially/ethnically representative sample of Arkansas during the COVID-19 pandemic. This compares to food insecurity rate of 13.8% in Arkansas prior to the pandemic [20]. Many of our findings are consistent with literature detailing the unequal odds of experiencing food insecurity before the pandemic. However, we contribute insights into how food insecurity relates to changes specific to the pandemic. We found that as respondents' age increased, odds of experiencing food insecurity decreased. Notably, this finding is supported by more recent studies of food insecurity amid the COVID-19 pandemic [12,21]. Consistent with prior research [12,[22], [23], [24]], we found racial and ethnic minorities, particularly Black and Hispanic/Latinx respondents, were two to three times more likely to report food insecurity compared with White respondents. Consistent with the extant literature [12,[24], [25], [26]], we found respondents who earned less than $49,999 were more likely to experience food insecurity relative to respondents earning over $75,000. We found households with children reported more food insecurity relative to households with no children, which is consistent with literature both before and during the COVID-19 pandemic [12,25,27,28]. While most children who are infected with COVID-19 have not experienced as severe health effects relative to adults [29], the COVID-19 pandemic's economic impact on households with children, and increases in food insecurity specifically, may have long-term health implications. Overall, these findings suggest that many of the groups who were vulnerable to food insecurity before the pandemic remain vulnerable during the pandemic.

Food insecurity was also associated with several factors specific to the COVID-19 pandemic, including income loss, not receiving a stimulus check, and changes in household size; however, change in household size was not significantly associated with food insecurity after controlling for other independent variables. Our measure of changes in household size was intended to broadly capture something about the disruptions in households that can occur during large-scale economic or natural disasters (e.g., doubling up with friends or family or decreases in household size due to death of a family member). COVID-19 has been credited for generating the highest rates of unemployment and income loss since the Great Depression [9]. Socially vulnerable groups including those who are lower-income and households with children have had the greatest income loss [11]. We found respondents who reported a loss of income due to COVID-19 were more likely to experience food insecurity. In the present study, 48.6% of households with children reported a loss of income, while 29.7% of those who did not have children reported a loss of income. Our findings are consistent with other research which documents large reductions in food insecurity associated with financial support (e.g., unemployment insurance) during the pandemic [30]. To address the economic hardships brought about by the COVID-19 pandemic, policymakers implemented The Coronavirus Aid, Relief, and Economic Security (CARES) Act. Although the CARES Act can be attributed with alleviating some financial stress brought on by the pandemic, research shows food insecurity continues to persist at extremely high levels [31]. Our findings demonstrate that Arkansas continued to experience high levels of food insecurity even after qualifying recipients received their stimulus check. However, those who reported that they received their stimulus check were less likely to be food insecure, suggesting that this policy was an important factor in reducing food insecurity for many Arkansans during this period of the pandemic. Overall, our findings provide valuable insights regarding the association between income loss, receipt of a stimulus check, and risk of food insecurity.

### 4.1 Limitations

This study has several limitations. First, the analysis is limited by the use of non-random sampling, and therefore, the findings may not be generalizable to the larger population. Although the data demonstrate levels of food insecurity above what has been previously reported for the state of Arkansas, our sample was over-representative of women and college graduates. Given that our sample includes more college graduates, who tend to experience less food insecurity than those with less education, our findings may underestimate the overall prevalence of food insecurity in the state. This limitation is countered somewhat by the similarity between the racial composition of our sample and that of the state population. Second, the study was limited by rapidly changing policy conditions. At the time we conducted our survey, eligible respondents had only received their first round of stimulus checks, and there have been two more rounds of stimulus checks distributed since the study was concluded. If the survey had been conducted earlier or later in the pandemic, the findings may have varied. Third, the data are cross-sectional and cannot establish causality. Further, there are many possible mechanisms through which the pandemic may be shaping food insecurity, and we were unable to assess many of them (e.g., food prices, shortages, or physical access). Finally, all responses are self-reported and do carry a risk of respondent bias. This limitation is reduced because of the large, diverse sample and the use of instruments validated for population survey research.

## 5 Conclusion

The present study found elevated food insecurity among a racially/ethnically representative sample of Arkansas during the COVID-19 pandemic. Food insecurity was elevated even after most respondents received a stimulus check and was particularly high among respondents who were younger, Black, Hispanic/Latinx, lower-income, less educated, and living in households with children. While several studies have documented increased food insecurity during the COVID-19 pandemic, this study contributes to a limited literature examining specific COVID-19-related factors associated with food insecurity. Our analysis indicated that financial factors specific to the COVID-19 pandemic (e.g., income loss, not receiving a stimulus check) are associated with higher odds of reporting food insecurity. Individuals who had not received a stimulus check may have still been awaiting their check or may have been ineligible for payment due to income level, being claimed as a dependent by a family member, or certain immigrant statuses. Despite the implementation of the CARES Act, the primary public policy solution aimed at reducing economic hardship brought about by the COVID-19 pandemic, food insecurity prevalence remained elevated well above state-level estimates for previous years. Our findings highlight the need for policy changes that are effective in providing socially vulnerable groups with the appropriate economic assistance they need during a public health crisis.

## Financial support

Support was provided by University of Arkansas for Medical Sciences Translational Research Institute funding through the National Center for Research Resources and National Center for Advancing Translational Sciences of the 10.13039/100000002National Institutes of Health (NIH) (UL1 TR003107). The NIH had no role in the design, analysis, or writing of this article.

## Authorship

Don E. Willis, Christopher R. Long, and Pearl A. McElfish formulated the research questions. Pearl A. McElfish and Christopher R. Long designed and carried out the study. Don E. Willis analyzed the data in consultation with Christopher R. Long. Don E. Willis, Caitlin Tidwell, Brett Rowland, and Jennifer A. Andersen wrote the initial draft of the article, followed by significant revisions from Christopher R. Long and Pearl A. McElfish.

## Ethical standards disclosure

This study was conducted according to the guidelines laid down in the Declaration of Helsinki, and all procedures involving research study participants were approved by the University of Arkansas for Medical Sciences (IRB #261226). Written informed consent was obtained from all subjects/patients.

## Declaration of competing interest

The authors declare that they have no known competing financial interests or personal relationships that could have appeared to influence the work reported in this paper.
